# Newly Generated Heparanase Knock-Out Mice Unravel Co-Regulation of Heparanase and Matrix Metalloproteinases

**DOI:** 10.1371/journal.pone.0005181

**Published:** 2009-04-10

**Authors:** Eyal Zcharia, Juan Jia, Xiao Zhang, Lea Baraz, Ulf Lindahl, Tamar Peretz, Israel Vlodavsky, Jin-Ping Li

**Affiliations:** 1 Department of Oncology, Hadassah-Hebrew University Medical Center, Jerusalem, Israel; 2 Department of Medical Biochemistry and Microbiology, Molecular Geriatrics, University of Uppsala, Uppsala, Sweden; 3 Department of Public Health and Caring Sciences, Molecular Geriatrics, University of Uppsala, Uppsala, Sweden; 4 Cancer and Vascular Biology Research Center, The Bruce Rappaport Faculty of Medicine, Technion, Haifa, Israel; Universität Heidelberg, Germany

## Abstract

**Background:**

Heparanase, a mammalian endo-β-D-glucuronidase, specifically degrades heparan sulfate proteoglycans ubiquitously associated with the cell surface and extracellular matrix. This single gene encoded enzyme is over-expressed in most human cancers, promoting tumor metastasis and angiogenesis.

**Principal Findings:**

We report that targeted disruption of the murine heparanase gene eliminated heparanase enzymatic activity, resulting in accumulation of long heparan sulfate chains. Unexpectedly, the heparanase knockout (*Hpse*-KO) mice were fertile, exhibited a normal life span and did not show prominent pathological alterations. The lack of major abnormalities is attributed to a marked elevation in the expression of matrix metalloproteinases, for example, MMP2 and MMP14 in the *Hpse*-KO liver and kidney. Co-regulation of heparanase and MMPs was also noted by a marked decrease in MMP (primarily MMP-2,-9 and 14) expression following transfection and over-expression of the heparanase gene in cultured human mammary carcinoma (MDA-MB-231) cells. Immunostaining (kidney tissue) and chromatin immunoprecipitation (ChIP) analysis (*Hpse*-KO mouse embryonic fibroblasts) suggest that the newly discovered co-regulation of heparanase and MMPs is mediated by stabilization and transcriptional activity of β-catenin.

**Conclusions/Significance:**

The lack of heparanase expression and activity was accompanied by alterations in the expression level of MMP family members, primarily MMP-2 and MMP-14. It is conceivable that MMP-2 and MMP-14, which exert some of the effects elicited by heparanase (i.e., over branching of mammary glands, enhanced angiogenic response) can compensate for its absence, in spite of their different enzymatic substrate. Generation of viable *Hpse*-KO mice lacking significant abnormalities may provide a promising indication for the use of heparanase as a target for drug development.

## Introduction

Studies on the involvement of extracellular matrix (ECM) molecules in cell attachment, growth and differentiation revealed a central role of heparan sulfate proteoglycans (HSPGs) in embryogenesis, morphogenesis, angiogenesis and epithelial-mesenchymal interactions [1], [2], [3]. Heparan sulfate (HS) chains interact with a multitude of proteins and ensure that a wide variety of bioactive molecules (e.g.-growth factors, chemokines, lipoproteins, enzymes) bind to the cell surface and ECM. HSPGs can thus influence a number of normal and pathological processes, among which are tissue repair, neurite outgrowth, inflammation and autoimmunity, tumor growth and metastasis, vasculogenesis and angiogenesis [1], [2], [3], [4], [5]. The ECM provides an essential physical barrier between cells and tissues, as well as a scaffold for cell growth, migration, differentiation and survival. It undergoes continuous remodeling during development and in certain pathological conditions such as wound healing, inflammation and cancer [6]. Cleavage of HS side chains is therefore expected not only to alter the structural integrity of the ECM, but also to release and modulate the activities of HS-bound biological mediators [4].

Mutational studies in drosophila and targeted disruption of murine genes involved in HS biosynthesis, or encoding HSPG core proteins, have demonstrated a critical role for HSPGs in developmental processes [7], [8], [9]. The observed phenotypes vary dramatically in severity, from failure to gastrulate [10] to selective impairment of mast cell function in otherwise seemingly healthy mice [11].

Heparanase is an endo-β-D-glucuronidase capable of cleaving HS side chains at a limited number of sites [12], [13], [14]. Heparanase activity has been correlated and causally associated with the metastatic potential of tumor-derived cells, attributed to enhanced cell dissemination as a consequence of HS cleavage and remodeling of the ECM barrier [14], [15]. Similarly, heparanase activity is implicated in neovascularization, inflammation and autoimmunity, involving migration of vascular endothelial cells and activated cells of the immune system [14], [15], [16]. Moreover, heparanase upregulation correlates with increased tumor vascularity and poor post-operative survival of cancer patients [17], [18], [19], [20].

The present study focuses on the generation of heparanase deficient mice by gene targeting. Despite complete lack of heparanase gene expression and enzymatic activity, the mice developed normally, were fertile and did not exhibit apparent anatomical and functional abnormalities, presumably because heparanase deficiency was compensated in most organs (i.e., liver, kidney) by a marked elevation in the expression of matrix metalloproteinase i.e., MMP-9 and MMP-14 family members. This newly discovered association between heparanase and MMPs suggests a combined interdependent control mechanism regulating the basal level of ECM degrading enzymes in cells and tissues.

## Materials and Methods

### Gene targeting construct

A 15-kb genomic clone containing the 5′ end of the heparanase gene (*Hpse*) was isolated from a bacteriophage mouse (strain 129/Sv) genomic library (Stratagene, Cedar Creek, TX). A 2.5-kb fragment upstream of exon 1 was cloned, as the short homologous arm, into pNT-Lox2 plasmid (kindly provided by Dr. Peter Carmeliet, Department of Molecular and Cellular Medicine, Catholic University, Leuven, Belgium) downstream of the neomycin resistance gene (*neo* cassette). A 4.8-kb fragment downstream of exon 1 was cloned upstream of the *neo* cassette as the long homologous arm of the endogenous gene. The targeting vector construct had a total size of about 14.5-kb.

### Homologous recombination in ES cells and generation of heparanase deficient mice

The targeting vector was linearized by the restriction enzyme Not I and electroporated into R1 embryonic stem (ES) cells. Clones expressing the *neo*-resistant gene were selected by including G418 (350 µg/ml; Invitrogen, Carlsbad, CA) in the cell culture medium and analyzed for target gene homologous recombination by Southern blot analysis of genomic DNA. The positive clones were injected into C57BL/6 blastocysts, and chimeric male founder mice were crossed with C57BL/6 females. Heterozygous mice were further crossed for 10 generations with C57BL/6J mice to produce *Hpse*-KO mice on a C57BL/6J background. All animal experiments were conducted in compliance with the Swedish and the Israeli legislation for animal welfare. All the animal experiments were approved by the animal committees of the Hebrew University, Jerusalem Israel (MD-89.49-4) and the Biomedical Center, University of Uppsala, Sweden (C176/2) according to the NIH guidelines.

### Genotype analysis

**Figure 1:**
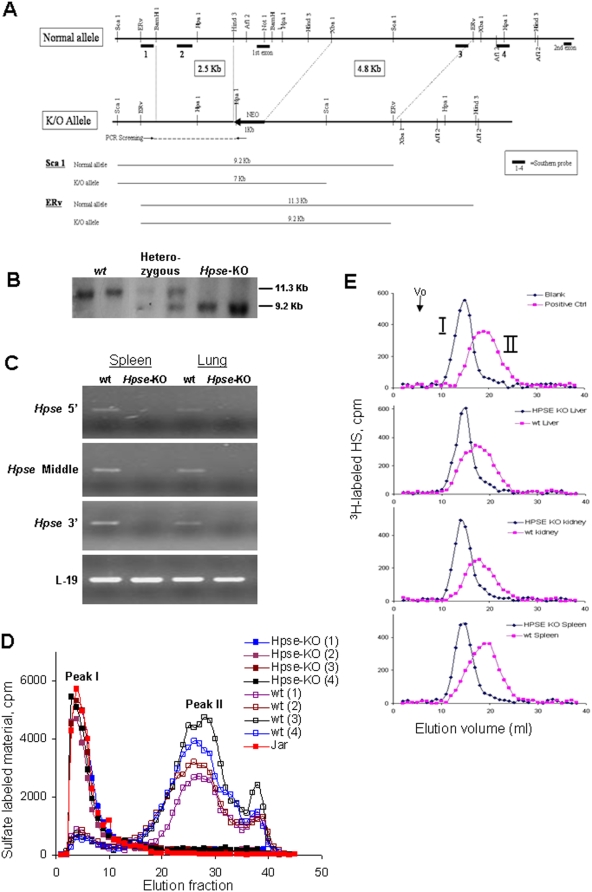
Targeted disruption of the heparanase gene and generation of heparanase deficient mice. A. Structure of the 5′ end of the heparanase (*Hpse)* gene (top-normal allele), the targeting vector (middle- K/O allele), and the expected size of products obtained by digestion with Sca1 or EcoRv (bottom). The orientation of the *neo* cassette and the southern blot probes are indicated. B. Southern blot analysis. Genomic DNA was extracted from embryos of the intercross of *Hpse* +/− heterozygous mice and subjected to Southern blot analysis after digestion with ERv. Wild type (*wt*) embryos exhibited only the normal allele, heterozygous embryos exhibited both the normal and the mutated allele, while *Hpse-*KO mice exhibited only the shorter, KO allele. Samples were hybridized with the external probe 3, shown in A. C. Heparanase mRNA expression. RNA was extracted from lungs and spleens of *wt* and *Hpse-*KO mice and subjected to PCR analysis of heparanase expression using 3 different PCR primer pairs designed to amplify different regions of the *Hpse* gene, as indicated. Heparanase expression was identified in samples derived from *wt* but not from *Hpse-*KO mice. D. Heparanase activity. Blood samples derived from 4 *wt* and 4 *Hpse-*KO mice, as well as total cell lysate derived from JAR cells, were incubated (16 h, 37°C, pH 6.2) with sulfate labeled ECM. Labeled degradation products released into the incubation medium were subjected to gel filtration analysis on Sepharose 6B, as described in “Materials and Methods”. High heparanase activity was noted only in samples derived from *wt* mice; no heparanase activity was detected in *Hpse-*KO samples or JAR cell lysate. E. HS degradation. Liver, kidney and spleen tissue extracts derived from *wt* and *Hpse-*KO mice were prepared as described in “Materials and Methods” and incubated (18 h, 37°C, pH 5.8) with ^3^H-acetyl labeled HS. The reaction mixture was then subjected to gel chromatography on Superose-12. The upper panel shows blank (peak I; control ^3^H-acetyl labeled HS substrate) and positive control (control substrate treated with recombinant heparanase; peak II). Incubations of *Hpse*-KO tissue extracts (black) resulted in the same elution profile as the blank incubation (upper panel), indicating no detectable heparanase activity. In contrast, incubations with *wt* tissue extracts (purple) resulted in substantial cleavage of the HS substrate, similar to incubation with recombinant heparanase (upper panel).

The genotype was determined by Southern blot analysis and PCR. Briefly, genomic DNA extracted from ES cells or tail biopsies was digested with either EcoR_V_ or Sca1. The resulting fragments were separated on 0.8% agarose gels and blotted onto a Nylon membrane, followed by hybridization with ^32^P-labeled probe #3, as shown in Figure 1A.

### Detection of gene expression

Total RNA was isolated from about 100 mg tissue using TRIzol (Invitrogen, Carlsbad, CA), according to the manufacturer's instructions, and quantified by spectrophotometry. After oligo (dT)-primed reverse transcription of 500 ng of total RNA, the resulting single stranded cDNA was amplified using the primers listed in Table 1. PCR conditions for heparanase were denaturation for 2 min at 94°C followed by 25 cycles of denaturation for 15 seconds at 94°C, annealing for 1 min at 58°C, and extension for 1 minute at 72°C. Aliquots (10 µl) of the amplified products were separated by electrophoresis on a 1.5% agarose gel and visualized by ethidium bromide staining (Hy Labs, Rehovot, Israel). The primers used for PCR are summarized in Table 1.

**Table 1:** PCR primers.

Gene	sequence (5′→3′)
L-19	S: 5′-ATGCCAACTCTCGTCAACAG-3′
	AS: 5′-GCGCTTTCGTGCTTCCTT-3′
*Hpse* 5′	S: 5′-CGACCGACGACGTGGTAGAC-3′
	AS: 5′-GCAACAGCTCCTGGAAGGG-3′
*Hpse* middle	S: 5′-TTTCTGAGCTCTGATGCGCTG-3′
	AS: 5′-TGGGCCTTTCACTCTTGACAG-3′
*Hpse* 3′	S: 5′-ACTTGAAGGTACCGCCTCCG-3′
	AS: 5′-GAAGCTCTGGAACTCGGCAA-3′

### Preparation of ECM coated dishes

Bovine corneal endothelial cells were established and cultured as described [21]. For preparation of sulfate-labeled ECM, corneal endothelial cells were cultured in the presence of Na_2_[^35^S]O_4_ (GE Healthcare Bioscience, Uppsala, Sweden) added (25 µCi/mL) on days 1 and 5 after seeding. Seven to 10 days later, the cell monolayer was dissolved and the ECM exposed, as described [21], [22].

### Heparanase activity

Heparanase enzymatic activity was determined in blood samples and tissue extracts derived from heparanase deficient (Hpse -KO) and wild type (*wt*) mice. Serum samples were diluted 1:1 in reaction buffer (20 mM phosphate–citrate buffer pH 6.2, containing 1 mM dithiothreitol, 1 mM CaCl_2_, and 50 mM NaCl) and incubated (16 h, 37°C) with ^35^S-labeled ECM, prepared as described above. The incubation medium was centrifuged (20,000×g, 4°C, 1 min), and the supernatant containing ^35^S-labeled HS degradation fragments was analyzed by gel filtration on a Sepharose CL-6B column. Fractions (0.2 mL) were collected and the amount of radioactivity in each fraction was counted in a beta scintillation counter. Nearly intact HSPGs were eluted from Sepharose 6B just after the void volume (peak I, Kav<0.2), while HS degradation fragments are eluted towards the V_t_ of the column (peak II, 0.5<kav<0.8) [4], [22], [23].

For analysis of heparanase activity in tissue samples, 4 month old *wt* and Hpse-KO mice were sacrificed, and the organs were immediately homogenized in 2 ml PBS, pH 7.4, containing 1% Triton X-100 and a protease inhibitor cocktail (Sigma-Aldrich, St. Louis, MO). The homogenates were incubated on ice for 30 min followed by centrifugation at 4°C, 15,000 rpm for 20 min. The supernatant was loaded onto a HiTrap heparin-Sepharose (GE Healthcare Bioscience) column equilibrated in the homogenization buffer. After washing with 10 ml PBS, the bound material was eluted with PBS containing 1M NaCl. The total amount of protein was determined by the Bradford method [24]. Samples of 50 µg protein from the elution were incubated (37°C, overnight) with 5,000 cpm [^3^H]acetyl-labeled HS in 20 mM phosphate-citrate buffer, pH 5.8, 1 mM dithiothreitol, 1 mM CaCl_2_, and 50 mM NaCl. The resulted products were analyzed by gel chromatography on Superose-12 column (GE Healthcare Biosciences).

### Metabolic radiolabeling and isolation of HSPG/HS

Wild type and *Hpse-*KO mice were injected intra-peritoneally with 0.5 mCi Na_2_^35^SO_4_ (specific activity 1,500 Ci/mmol; Perkin Elmer, Waltham, MA) and maintained for 45 min with free access to water and food. The animals were then sacrificed by cervical dislocation and the organs were dissected. The tissues were homogenized with a Dounce homogenizer in 6 volumes of ice-cold 50 mM Tris-HCl, pH 7.4, 1% (v/v) Triton X-100, 4 M urea, 0.25 M NaCl containing a protease inhibitor cocktail (Sigma-Aldrich), followed by incubation at 4°C overnight. Following centrifugation, the supernatants were applied to DEAE-Sephacel columns equilibrated in 50 mM Tris-HCl, pH 7.4, 0.3 M NaCl. The columns were extensively washed with the same buffer and were then eluted with the same buffer containing 1.5 M NaCl. Eluates were desalted, lyophilized and digested with chondroitinase ABC (Seikagaku, Tokyo, Japan) and benzonase (Merck, San Diego, CA). The digests were then re-applied to DEAE-Sephacel to remove degraded chondroitin sulfate and oligonucleotides [25]. The HSPG fractions eluted with 1.5 M NaCl were pooled for further analysis.

### Characterization of HS molecular structure

Gel chromatography of HSPGs and HS free chains was preformed on a Superose 12 column eluted with 50 mM Tris-HCl, pH 7.4, 1 M NaCl, 0.1% Triton X- 100. To analyze domain organization, HS samples were subjected to cleavage at N-sulfated GlcN residues by treatment with nitrous acid at pH 1.5, followed by reduction with NaB[^3^H]_4_ [25]. The reduced products were separated by gel chromatography on a column (1×200 cm) of Bio-Gel P-10 (Bio-Rad, Hercules, CA) in 0.5 M NaCl. A portion of the nitrous acid degradation products was applied to a Sephadex G-15 column and disaccharides were recovered. After desalting and concentration, the ^3^H or ^35^S labeled disaccharides, derived from *N*-sulfated domains, were further analyzed by anion-exchange HPLC on a Partisil-10 SAX column, as previously described [8].

### Biochemical analysis

Urine samples (25 µl) were analyzed for total protein and creatinine content, using the automated Kodak 250 system [26]. Blood samples (25 µl) were examined for creatinine, aspartate aminotransferase (AST), alanine aminotransferase (ALT), and alkaline phosphatase (ALP) content, using an automated Kodak 950 system [26].

### Mammary gland whole mount histostaining

Whole-mount mammary glands, prepared as described [27], were fixed in Tellys fixative (100 ml EtOH 70%, 5 ml formalin, 5 ml glacial acetic acid), rehydrated and stained with hematoxylin for 3 h. After staining, the glands were washed in tap water (1 h), dehydrated and stored in methyl salicylate.

### Matrix metalloproeinase analysis

Real-time quantitative PCR analysis was performed with an automated rotor gene system RG-3000A (Corbett research, Sydney, Australia). The PCR reaction mix (20 µl) was composed of 10 µl QPCR sybr master mix (finnzymes, Espoo, Finland), 5 µl of diluted cDNA (each sample in a six-plicate) and a final concentration of 0.3 µM of each primer. PCR conditions were as follows: an initial denaturation step at 95°C for 15 min; 40 cycles of denaturation at 94°C for 15 s, hybridization at 57°C for 30 s, and elongation at 72°C for 30 s. The primers used for this reaction are summarized in Table 2. Actin primers were used as an internal standard. The expression level of different MMPs in the *wt* tissue was regarded as 100%, and the MMP levels in the *Hpse-*KO mice were calculated relative to this value.

**Table 2:** Real Time PCR primers.

Gene	sequence (5′→3′)
Human	
Actin	S: 5′-TCCCTGGAGAAGAGCTACG-3′
	AS: 5′-GTAGTTTCGTGGATGCCACA-3′
*Hpse*	S: 5′-TACCTTCATTGCACAAACACTG-3′
	AS: 5′-ACTTGGTGACATTATGGAGGTT-3′
MMP2	S: 5′-GCGGCGGTCACAGCTACTT-3′
	AS: 5′-CACGCTCTTCAGACTTTGGTTCT-3′
MMP9	S: 5′-CCTGGAGACCTGAGAACCAATC-3′
	AS: 5′-CCACCCGAGTGTAACCATAGC-3′
MMP14	S: 5′-CGCTACGCCATCCAGGGTCTCAAA-3′
	AS: 5′-CGGTCATCATCGGGCAGCACAAAA-3′
MMP25	S: 5′-AGTTGCTGTCCAGCCTCAGT-3′
	AS: 5′-CCAAAGTCTCCTGCCTTCTG-3′
Mouse	
Actin	S: 5′-ATGCTCCCCGGGCTGTAT-3′
	AS: 5′-CATAGGAGTCCTTCTGACCCATTC-3′
MMP2	S: 5′-AGCGTGAAGTTTGGAAGCAT-3′
	AS: 5′-CACATCCTTCACCTGGTGTG-3′
MMP9	S: 5′-AGACGACATAGACGGCATCC-3′
	AS: 5′-GTGGTTCAGTTGTGGTGGTG-3′
MMP14	S: 5′-GCCTGGAACATTCTAACGA-3′
	AS: 5′-CTTTGTGGGTGACCCTGACT-3′
MMP25	S: 5′-GCTGACTCGCTATGGCTACC-3′
	AS: 5′-GTCATTGGGTCCATTTGTCC-3′
Hpa2	S: 5′-GTCCGTGGCTCTATCACACTTT-3′
	AS: 5′-CCTCAGAGTCCCAGCCAGTTT-3′

### Western blot analysis

For immunoblot analysis, aliquots of tissue extracts (50 µg) were separated by electrophoresis in 10% SDS-polyacrylamide gel (PAGE) and transferred to Immobilon-P membrane (Millipore, Bedford, MA). MMP2 was detected by anti-mouse MMP2 monoclonal antibodies 801B (1:150, kindly provided by Dr. Rafael Fridman, Wayne State University, Detroit, MI). β-catenin was detected by anti-mouse monoclonal antibody (1:150, BD transduction laboratories, San Jose, CA), and anti mouse α-tubulin clone B-5-1-2 (1:5000; sigma). Membranes were incubated with primary antibodies for 2 h at room temperature, washed in TTBS and probed with HRP-conjugated secondary antibody (Jackson Laboratories, Bar Harbor, ME). After several washes in TTBS, detection of the secondary antibodies was performed using the SuperSignal Chemiluminescent Substrate system (Pierce, Rockford, IL). The chemical illumination signals were exposed to Fuji medical X-ray film (Super RX).

### Aortic ring assay

Aortic ring assay was performed as previously described [28]. Briefly, 1 mm mouse aortic rings were embedded in 3-dimensional growth factor depleted Matrigel (BD Biosciences, San Jose, CA), and incubated in 0.5 ml Bio-MPM (Biological industries, Beit haemek, Israel) in the absence or presence of added FGF-2 (50 ng/mL). The rings were maintained for 6 days (37°C, 8% CO_2_, humidified atmosphere), and both the medium, and FGF-2 were replaced every 2 days. Vascular sprouting was evaluated every day for a period of 6 days, then fixed with 4% formalin for 24 h and stained with 0.02% crystal violet in ethanol (Sigma-Aldrich), and photographed using a Nikon Eclipse TS 100 phase-contrast microscope.

### Matrigel plug assay

Matrigel plug assay was performed as described [29]. Briefly, mice were injected subcutaneously with 200 µL of growth factor depleted Matrigel, supplemented with FGF-2 (80 ng/mL). Seven days later, the Matrigel plugs were excised, photographed, homogenized in a hypotonic lysis buffer (250 µl of 0.1% Brij-35/plug) and centrifuged for 5 min at 5,000 g. The supernatant was used in duplicates to measure hemoglobin with Drabkin's reagent [30].

### Chromatin immunoprecipitation

Chromatin Immunoprecipitation assay was performed as described [31]. Briefly, cross-linking between DNA and protein was preformed by adding formaldehyde (Merck) directly into the culture medium of mouse embryonic fibroblasts (MEF) derived from *wt* and *Hpse*-KO mice. After fixation and lysis, DNA pellets were sonicated into chromatin fragments of an average length of <500 bp. ChIP was performed with anti-β-catenin monoclonal antibody (1:150, BD transduction laboratories, San Jose, CA) preincubated with magnetic bead-conjugated mouse immunoglobulin G (IgG) (Dynabeads M-280 sheep anti-mouse IgG, Dynal Biotech ASA) at 4°C, overnight with rotation. Elution of extensively washed immune complexes was carried out by the addition of 50 µL of elution buffer [50 mmol/L Tris-HCl (pH 8), 10 mmol/L EDTA, 1% SDS] at 65°C for 15 min with brief vortexing every 2 min [31]. Reverse cross-linking was carried out by incubating at 65°C for overnight. RNA and unbound proteins were removed by the addition of 0.2 mg/mL RNase A for 1 h at 37°C, followed by the addition of 0.2 mg/mL proteinase K for 2 h at 55°C. Recovered chromatin was suspended in 50 µL TE buffer [31]. Quantitative PCR analysis was performed using 5 µL of immunoprecipitated chromatin or input chromatin using Dynamo sybr master mix (Finnzymes, Espoo, Finland). Amplifications were done using specific primer sets flanking the putative Lef/Tcf motifs in the MMP-14 promoter. MMP14-Pro1: sense 5′-ttccccacccccacacacaa-3′, antisense 5′-caggaataaagccaggtaggaa-3′, MMP14-Pro2: sense 5′-ctacccaggcagttcacactt-3′, antisense 5′-caagagggttgtatgctgaga-3′.

### Statistical Analysis

InStat statistical software, version 3.05 (GraphPad Software, San Diego, CA), was used to calculate statistical differences between groups (tested by the paired Student's *t* test). All *P* values were two-sided.

## Results

### Targeted disruption of the heparanase (*Hpse*) gene

We have generated heparanase deficient mice through targeted interruption of the gene in ES cells. A targeting vector was constructed to create a functional mutation by deleting the minimal promoter region (about 500 bp upstream of the transcription start point) and the entire first exon (Fig. 1A). Following electroporation, screening of 400 *neo*-resistant ES clones resulted in two homologous recombinations in the *Hpse* gene, identified by Southern blot analysis, with no additional integration sites. Microinjection of both clones into C57BL/6 blastocysts yielded chimeric animals, one of which showed germ line transmission. Breeding of the chimeric mouse with C57BL/6 strain yielded heterozygous mice which did not show any overt defects.

Genotypic analysis of offsprings from intercrosses between heterozygous littermates (Fig. 1B) showed essentially Mendelian heritance, indicating no early embryonic death. To confirm complete interruption of the *Hpse* gene, we examined the expression of heparanase mRNA derived from different tissues of *wt* and *Hpse*-KO mice by real time PCR using specific primers as summarized in Table 1. The primers were designed to amplify the 5′, middle, and 3′ regions of the heparanase gene. As shown in figure 1C, heparanase mRNA was detected only in samples derived from *wt* mice, but not in samples from the *Hpse*-KO mice.

To verify the elimination of heparanase enzymatic activity, we analyzed 4 blood serum samples and 3 tissues from *wt* and *Hpse-*KO mice using sulfate labeled intact ECM (Fig. 1D) or soluble HS side chains (Fig. 1E) as substrates. Regardless of the assay system, none of the samples from *Hpse-*KO mice showed heparanase activity, while the *wt* samples exhibited normal levels of heparanase activity (Fig. 1D and E). The degradation pattern exhibited by *Hpse-*KO derived serum samples is identical to that demonstrated by JAR human choriocarcinoma cells (Fig. 1D) shown to have no heparanase activity due to extensive promoter hypermethylation [32]. Of particular significance is the lack of heparanase activity in serum samples containing activated platelets and white blood cells known to express exceedingly high levels of the enzyme [33]. As demonstrated in figure 1D, blood samples derived from *wt* mice exhibited high levels of heparanase activity, as detected by the large amount of low molecular weight material eluted in fractions 20–40 (Fig. 1D, peak II). Labelled fragments eluted in peak II were shown to be degradation products of HS, as they were 5–6 fold smaller than intact HS chains of HSPGs, resistant to further digestion with papain and chondroitinase ABC, and susceptible to deamination by nitrous acid [34]. In contrast, blood samples derived from *Hpse-KO* mice exhibited no heparanase activity as revealed by the lack of HS degradation fragments (peak II) and the release of high molecular weight material eluted in fractions 5–10 (peak I, Fig. 1D). This material, representing nearly intact HSPGs, is produced by proteolytic cleavage of the proteoglycan core protein by proteases residing in the ECM and cell lysate [35]. Similarly, there was no cleavage of ^3^H-labeled HS upon incubation with extracts of liver, kidney and spleen derived from *Hpse*-KO mice (peak I) vs. a significant degradation of HS upon incubation with the corresponding *wt* tissue extracts (peak II, Fig. 1E). Altogether, these data clearly demonstrate complete elimination of heparanase enzymatic activity in the *Hpse*-KO mice.

### Phenotypic analysis of the *Hpse-*KO mice

The homozygous mutant animals showed no obvious aberrant phenotype, were fertile and exhibited a normal life span. To examine any possible age-related phenotypes, 6-, 12- and 18-month old mice were sacrificed and organs were dissected and fixed in a solution containing 96% ethanol, 1% glacial acetic acid and 3% distilled water. Paraffin embedded tissue sections were stained with hematoxilin and eosin. Histological examination of sections derived from the brain, heart, liver, lung, kidney and spleen did not reveal significant structural or pathological abnormalities in the *Hpse*-KO mice (not shown).

### Molecular structure of HS

**Figure 2:**
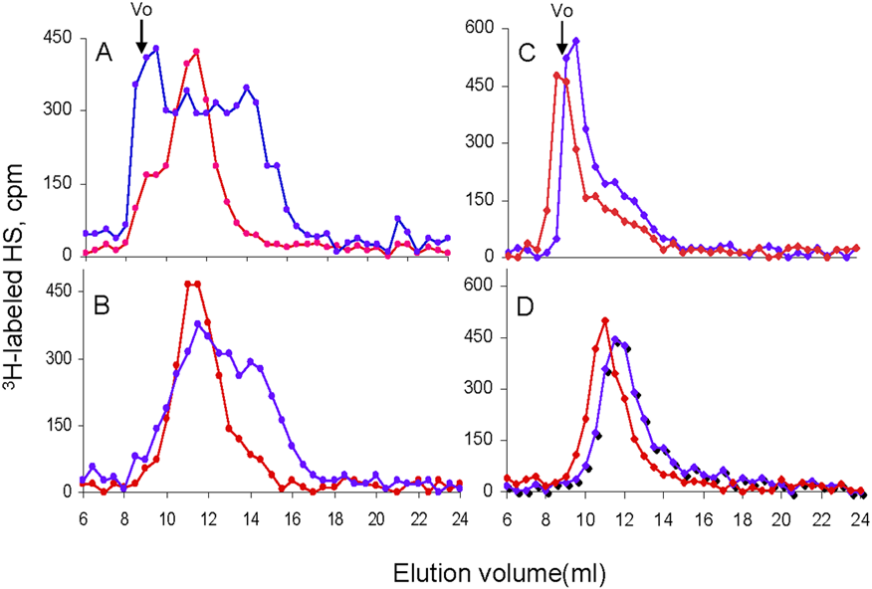
Molecular structure of HS from *wt vs. Hpse-*KO mice. Total metabolically ^35^S-labeled HSPGs was isolated as described in “Materials and Methods”. The samples were analyzed on a Superose 12 column as shown for liver (A) and kidney (C). The HSPGs were treated with alkali and the released free HS chains were analyzed on the same column as show for liver (B) and kidney (D). (Blue- *wt*; red- *Hpse*-KO). Standard heparin is eluted at a volume of 14 ml.

As degradation of HS is the major function of heparanase, we first examined the size of HS derived from selected organs. For this purpose, adult mice were metabolically labeled with Na_2_^35^SO_4_ and total HS was isolated and subjected to gel filtration chromatography. As demonstrated in figure 2, HS chains from *Hpse*-KO liver (Fig. 2A, B) and kidney (Fig. 2C, D) were of higher molecular mass in comparison to HS extracted from *wt* tissues (Fig. 2). In addition, the elution peaks of free HS chains isolated from *Hpse*-KO tissues appeared narrower and more symmetrical (Fig. 2B,D), indicating less heterogeneity in size distribution in comparison with the elution profile of HS side chains isolated from *wt* tissues (the overall broad size of peaks reflects the state of HS biosynthesis). Structural analysis of HS sulfation and disaccharide composition did not show a detectable difference between samples derived from *wt* and *Hpse-*KO tissues (not shown).

### Biochemical analysis

To examine the role of heparanase and effect of HS structural alternations on liver and kidney function, blood samples were taken from 15 *wt* and 15 *Hpse-*KO mice, before and after a 72h fasting. Samples were analysed for total protein and contents of creatinine, aspartate aminotransferase (AST), alanine aminotransferase (ALT), and alkaline phosphatase (ALP). No significant differences between *wt* and *Hpse-*KO mice were detected, both before and after fasting (data not shown). Furthermore, since platelets contain high amount of heparanase, blood samples were also examined for coagulation properties (i.e., APTT). Again, there was no significant difference between the *Hpse-*KO and *wt* mice (not shown).

### Mammary gland morphogenesis

**Figure 3:**
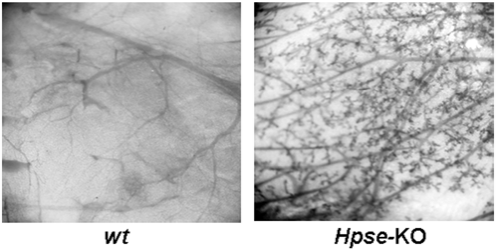
Morphological appearance of mammary glands from *wt* vs. *Hpse*-KO mice. Whole-mount preparations of mammary glands from 3-month-old virgin mice were stained with hematoxylin. *Hpse-*KO derived mammary glands (right panel) showed abundant side branches and alveolar structures compared with glands from age-matched *wt* animals (left panel).

We have previously reported that transgenic virgin mice over-expressing the human heparanase gene (*hpa*-tg mice) exhibited abnormal abundant branching of ducts in the mammary gland and precocious alveolar structures, typical of pregnant mice [36]. Notably, these differences decreased during late stages of pregnancy and during involution and did not affect the function of the mammary gland. To our surprise, a similar morphology was noted in the mammary glands of 3-month old virgin homozygous *Hpse-*KO mice (Fig. 3, right panels), as compared to poorly developed mammary glands seen in the *wt* virgin mice (Fig. 3, Left panels). However, unlike the *hpa-*tg vs. control mice [36], there was no significant difference between the *wt* and *Hpse*-KO mice in the width of the primary ducts.

### Endothelial sprouting and angiogenesis

**Figure 4:**
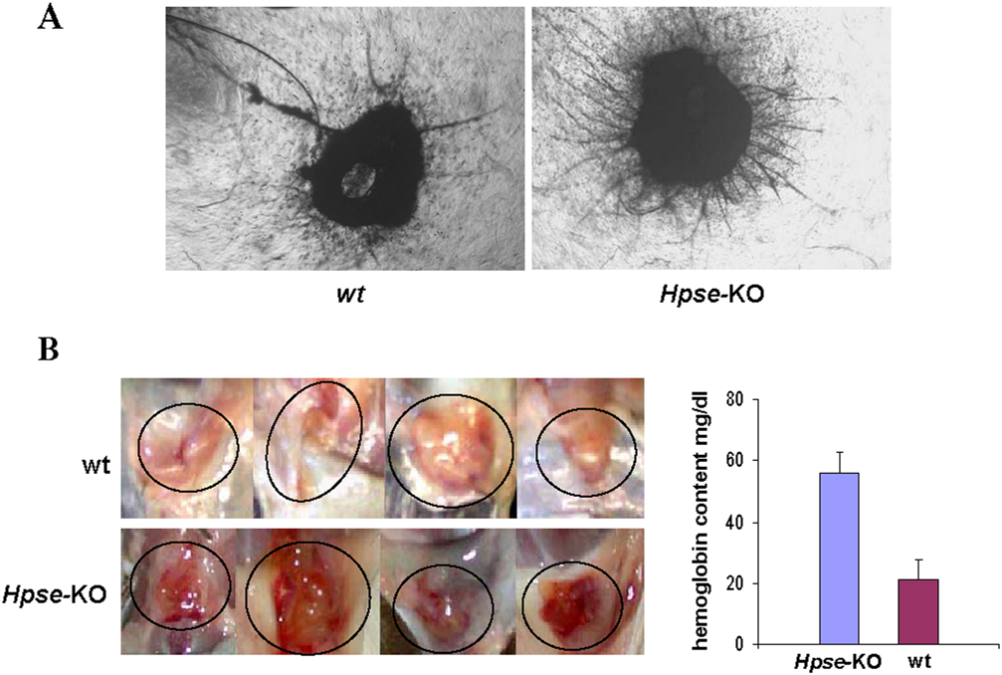
Endothelial sprouting and angiogenesis. A. FGF-2 induced vascular sprouting in the aortic ring model. *Hpse-*KO and *wt* derived aortic rings were subjected to FGF-2 induced vascular sprouting for 6 days. The rings were then fixed, stained with 0.02% crystal violet and evaluated for vascular sprouting. A more extensive endothelial sprouting was noted in *Hpse-*KO derived rings (right panel) as compared to *wt* derived rings (left panel). B. Matrigel plug assay. *Hpse-*KO (lower panels) and *wt* (upper panels) mice were injected subcutaneously with 200 µl of growth factor depleted Matrigel supplemented with FGF-2 (80 ng/ml). Seven days later, the Matrigel plugs were excised and photographed, followed by homogenization and determination of hemoglobin content using Drabkin's reagent (right). A pronounced angiogenic response was noted in *Hpse-*KO vs. *wt* mice (55±7.18 mg/dl vs. 21±6.2 mg/dl; p≤0.0002, respectively).

The involvement of heparanase in cell migration and angiogenesis is well documented [19]. We have, therefore, evaluated the effect of heparanase knockout on endothelial cell migration and sprouting. First, we applied an *ex vivo* aortic ring assay. Briefly, 8 *wt* and 8 *Hpse*-KO mice were sacrificed and their aortas were cleaned and cut into 1–2 mm thick rings. The rings were embedded in growth factor-depleted Matrigel and examined for endothelial sprouting upon simulation with FGF-2 (50 ng/ml). As expected, in the absence of FGF-2 there was little or no sprouting in either the *wt* or *Hpse-*KO rings (not shown). Upon stimulation with FGF-2, both *wt* and *Hpse*-KO rings showed endothelial sprouting (Fig. 4A). Notably, *Hpse-*KO derived rings exhibited a more pronounced sprouting (Fig. 4A, right panels) compared to *wt* aortic rings (Fig. 4A, left panels), suggesting an increased response to FGF-2 stimulation.

To validate the *ex vivo* results, we performed an *in vivo* angiogenesis assay. For this purpose, Matrigel supplemented with FGF-2 was implanted subcutaneously and formation of a capillary network within the Matrigel implants was visualized. Seven days after implantation, mice (n = 8) were sacrificed and the plugs were evaluated for neovascularization by measuring the content of hemoglobin in the Matrigel plug. A profound angiogenic response was induced by Matrigel-embedded FGF-2 in the *Hpse-*KO mice (Fig. 4B, bottom) compared with *wt* mice (Fig. 4B, top), corroborating the *ex-vivo* results. Determination of hemoglobin revealed a 2.6-fold increase in the hemoglobin content of Matrigel plugs embedded in *Hpse*-KO compared to *wt* mice (55.75±7.18 mg/dl vs. 21±6.25 mg/dl; p≤0.0002, respectively) (Fig. 4B, right).

### Interrelation between heparanase and MMPs

**Figure 5:**
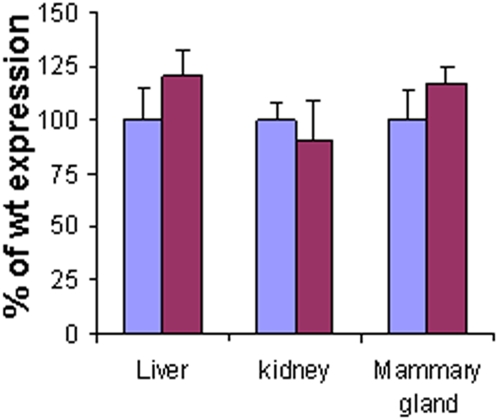
Hpa2 expression. RNA samples derived from liver, kidney and mammary glands of *wt* and *Hpse-*KO mice were subjected to quantitative real time PCR analysis to evaluate the expression of Hpa2. The expression level determined for each MMP in the *wt* tissue (blue) was regarded as 100% and the corresponding expression level determined in the *Hpse-*KO tissue (purple) is presented as percentage relative to the 100% value. Each reaction was repeated 6 times and the mean±SD is indicated. No significant difference was detected in Hpa2 expression between *wt* and *Hpse-*KO mice.

**Figure 6:**
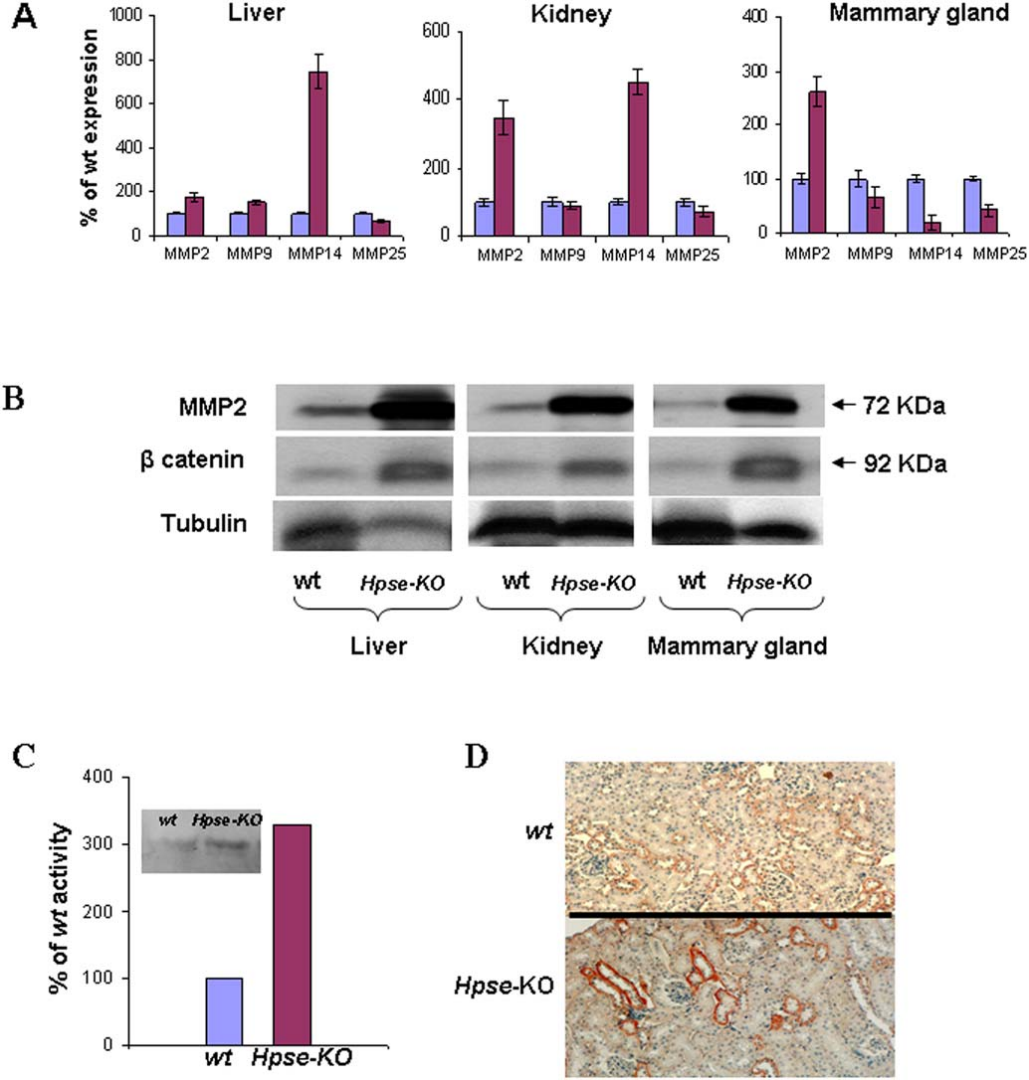
MMP expression in *Hpse-*KO mice. A. Real-time PCR. RNA was extracted from liver, kidney and mammary gland of *wt* and *Hpse-*KO mice and subjected to quantitative real time PCR analysis to evaluate the expression of MMP-2, MMP-9, MMP-14 and MMP-25. The expression level determined for each MMP in the *wt* tissue (blue) was regarded as 100% and the corresponding expression determined in the *Hpse-*KO tissue (Purple) are presented as percentage relative to it. Each reaction was repeated 6 times and the mean±SD is indicated. B. Western blot analysis. Liver, kidney and mammary gland tissue extracts, prepared as described in “Materials and Methods”, were subjected to Western blot analysis using anti-mouse MMP-2 monoclonal antibodies (mA801B; upper panels), anti mouse β-catenin (mAb610154; middle panels), or anti mouse α-tubulin (B-5-1-2; lower panels). Higher protein levels of MMP-2 and β-catenin were detected in samples derived from *Hpse-*KO vs. *wt* tissues. C. MMP2 zymography. Serum samples derived from *Hpse-*KO and *wt* mice were evaluated for MMP-2 activity. MMP-2 activity was approximately 3 fold higher in plasma samples derived from *Hpse-*KO vs. *wt* mice. D. β-catenin immunostaining. Parafin embedded kidney tissue sections were subjected to immunostaining with antibody directed against β-catenin. Increased staining was observed in kidney derived from *Hpse-*KO vs. *wt* mice. C. MMP2 zymography. Serum samples derived from *Hpse-*KO and *wt* mice were evaluated for MMP-2 activity. MMP-2 activity was approximately 3 fold higher in plasma samples derived from *Hpse-*KO vs. *wt* mice.

The unexpected result of abnormal mammary gland morphology and increased neovascularization in *Hpse-*KO mice led us to search for a possible explanation for this phenotype. The immediate question was whether other ECM degrading enzyme(s) were compensating for the lack of heparanase expression. *Hpa*2, a gene exhibiting significant homology (∼38%) to the heparanase gene [37], but lacking a detectable heparanase enzymatic activity, was the first candidate to examine. For this purpose, total RNA extracted from the kidney, liver and mammary gland of *Hpse-*KO and *wt* mice, was analyzed using specific primers corresponding to *Hpa*2 (Table 2). Analysis of *Hpa*2 expression in the different tissues revealed no significant difference between *wt* and *Hpse*-KO mice (Fig. 5). These results were further corroborated by Western blot of *Hpa*2 showing no difference in protein level between *Hpse*-KO and *wt* mice (not shown). Moreover, the increased HS length found in *Hpse-*KO mice does not support upregulation of additional heparanase-like enzyme. Taking into account that matrix metalloproteinases (MMPs) play important roles in rearranging the ECM structure and thereby in tissue remodeling, morphogenesis and neovascularization, we investigated the expression of MMPs (real-time PCR) in the *Hpse*-KO vs. *wt* mice. For this purpose, the RNA samples were further analyzed using specific primers corresponding to MMP-2, -3, -9, -14 and -25 (Table 2). The results (Fig. 6A) indicated that the lack of heparanase expression was associated with marked changes in the expression levels of several members of the MMP family. MMP-2 was over-expressed (2–3.5 fold) in all samples extracted from *Hpse-*KO vs. *wt* mice. MMP-14 was over-expressed (4–7 fold) in the liver and kidney, but down regulated (∼4 fold) in mammary glands derived from *Hpse-*KO vs. *wt* mice. MMP-9 and MMP-25 expression levels were altered, albeit to a lower extent, depending on the tissue (Fig. 6A).

These results were further corroborated by Western blot analysis revealing increased levels of the MMP-2 protein in homogenized samples extracted from the liver, kidney and mammary glands of *Hpse-*KO vs. *wt* mice (Fig. 6B). We also investigated whether the increased expression of MMP-2 is manifested by elevated MMP-2 enzymatic activity. For this purpose, plasma samples derived from *wt* and *Hpse-*KO blood were subjected to zymography and evaluated for MMP-2 activity. MMP-2 activity was approximately 3 fold higher in plasma samples derived from *Hpse-*KO vs. *wt* mice (Fig. 6C).

Next, we sought to investigate a possible molecular pathway involved in the interaction between heparanase and MMPs. Since β-catenin was previously implicated in MMP regulation [38], [39], we evaluated its content and staining pattern in tissues derived from *wt* and *Hpse-*KO mice by examining β-catenin accumulation. Western blot analysis of tissue extracts revealed accumulation of β-catenin in the soluble fraction of liver, kidney and mammary glands derived from *Hpse-*KO compared to *wt* mice (Fig. 6B, middle panel). Similarly, immunostaining of kidney tissue sections derived from *wt* and *Hpse-*KO mice (Fig. 6D) revealed a marked increase in β-catenin staining in *Hpse-*KO kidneys, corroborating the Western blot results.

**Figure 7:**
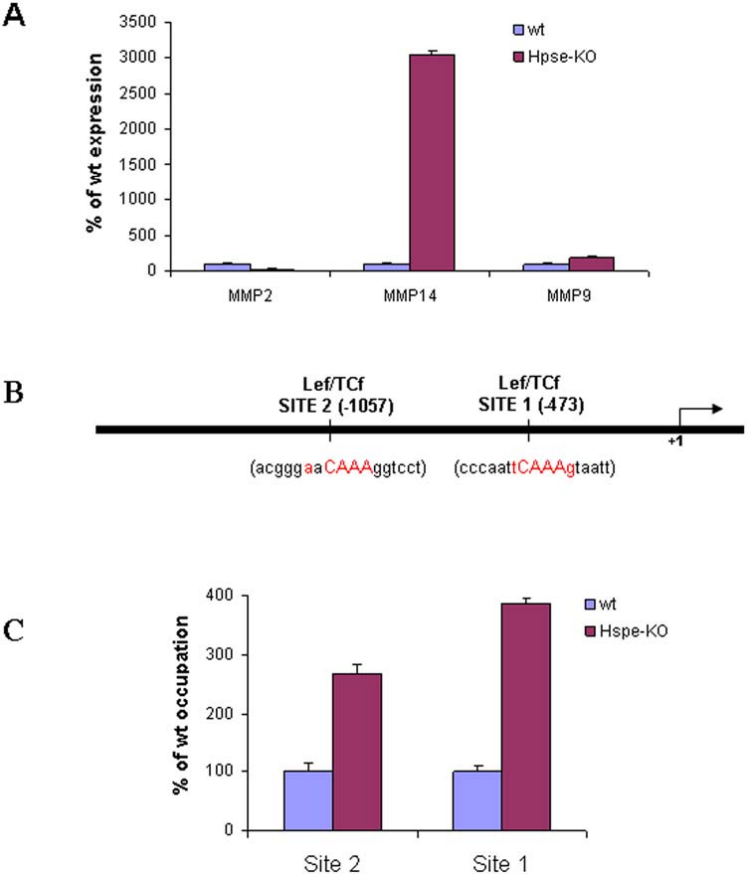
MMP expression and ChIP analysis for β-catenin in MEF cells derived from *Hpse-*KO and *wt* mice. A. Real-time PCR. RNA was extracted from MEF derived from *wt* and *Hpse-*KO mice and subjected to quantitative real time PCR analysis to evaluate the expression of MMP-2, MMP-9, and MMP-14. The expression level determined for each MMP in the *wt* tissue (blue) was regarded as 100% and the corresponding levels determined in the *Hpse-*KO cells (purple) are presented as percentage relative to it. Each reaction was repeated 6 times and the mean±SD is indicated. B. Schematic representation of regions along the murine MMP-14 promoter with ≥95% homology to the consensus Lef/Tcf motif. Numbers show the location of putative Lef/Tcf motifs relative to the transcription initiation site (*bent arrow*). C. ChIP analysis. Following cross-linking of proteins to DNA, chromatin derived from *Hpse-*KO and *wt* MEF was sonicated into fragments of average length ≤500 bp; the β-catenin protein was immunoprecipitated with anti β-catenin antibody, and PCR analysis was performed on the immunoprecipitated DNA samples using primer sets MMP14-Pro1 & Pro2, as described in ‘Materials and Methods’. Samples were equilibrated for DNA loading amounts using primers specific to actin. The results are representative of three independent experiments.

The increased levels of MMPs and β-catenin prompted us to apply chromatin immunoprecipitation (ChIP) analysis to test whether β-catenin binds directly to the MMP promoter. For this purpose, murine embryonic fibroblasts (MEF) derived from *Hpse-*KO and *wt* mice were first examined for MMP expression. As demonstrated in figure 7A, MMP-9 was 2 fold over expressed and MMP-2 was 4-fold under expressed in *Hpse-*KO compared to *wt* derived MEF. MMP-14 exhibited a marked 30 fold increased expression in *Hpse-*KO MEF and thus was chosen for ChIP analysis. Matinspector 2.2 promoter analysis software [40] revealed two putative binding sites for the Lef/Tcf motif in the murine MMP-14 promoter, at positions −473 and −1057 from the transcription starting point (Fig. 7B). Following chemical cross-linking, chromatin was isolated from *Hpse-*KO and *wt* MEF, as described in ‘Materials and Methods’. The cross-linked chromatin was sonicated to ≤500-bp fragments and immunoprecipitated with anti β-catenin antibody. DNA obtained from the immunoprecipitated chromatin was amplified using two sets of MMP-14 promoter-specific primers (MMP14-Pro 1 & 2), flanking the two putative Lef/Tcf motifs (Fig. 7B), as well as a set of primers specific to the unrelated actin gene sequence, to normalize for equal chromatin amounts. Quantitative real time PCR revealed a 3–4 fold enrichment of MMP-14 promoter sequences in the two Lef/Tcf sites of *Hpse-*KO vs. *wt* MEF (Fig. 7C). No enrichment was observed when antibody directed against an irrelevant protein (Flt-1 tyrosine kinase receptor) was used for the ChIP analysis (data not shown). These results indicate direct binding of β-catenin to MMP-14 gene regulatory sequences.

**Figure 8:**
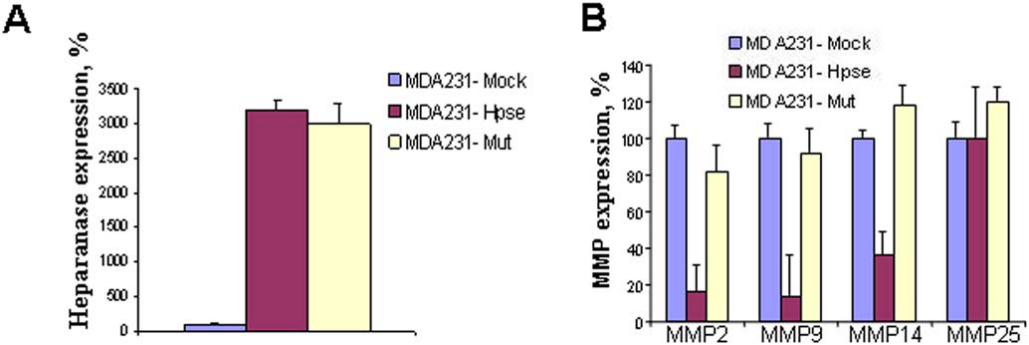
Expression of MMPs in heparanase transfected MDA-231 human breast carcinoma cells. MDA-MB-231 cells were transfected with a mock (empty vector) or either active (*Hpse*) or mutated inactive (Mut) heparanase gene. Heparanase (A) and MMPs (B) mRNA expression levels were determined by real-time PCR, as described under “Materials and Methods”. The appropriate primers are listed in Table 2. The expression levels determined in the mock transfected cells were regarded as 100%, and the levels in *Hpse* and mut-*Hpse* transfected cells were presented as percentage relative to the mock transfected cells. Decreased levels of MMP-2, MMP-9, and MMP-14 mRNAs were noted in cells over-expressing the active form of heparanase, but not the double mutant, inactive form of the enzyme.

To further investigate the interrelation between heparanase and MMPs, we transfected human breast carcinoma MDA-NB-231 cells, normally expressing moderate levels of heparanase [22], with either active or double mutant heparanase (active site Gln 225 and Gln 343 replaced by Ala) lacking enzymatic activity [41]. Both, the active and inactive mutant heparanase were ∼30 fold over-expressed in the *Hpse*-transfected cells as compared to mock transfected cells (Fig. 8A). As demonstrated in Fig. 8B, cells transfected with active heparanase exhibited a marked decrease in expression of MMP-2 (5.8 fold), MMP-9 (6.5 fold) and MMP-14 (3 fold), a mirror image of the increased expression found in *Hpse-*KO mice. In contrast, transfecting the MDA-231 cells with the double mutant inactive heparanase did not affect MMP expression (Fig. 8B), indicating that heparanase enzymatic activity is involved in the observed regulation of MMP expression.

## Discussion

HS glycosaminoglycans bind to and assemble ECM proteins (i.e., laminin, fibronectin, collagen type IV) and thereby contribute significantly to the self assembly and integrity of the ECM. HS also play important roles in cell-cell and cell-ECM interactions [2], [42], [43], [44], as well as in physiological and pathological processes such as tissue morphogenesis and repair, vascularization and cancer metastasis [1], [2], [3]. Over expression of heparanase, the predominant HS degrading enzyme, in transgenic mice and tumor tissues resulted in pronounced structural alterations of HS [25]. Heparanase is therefore expected to exert significant effects on embryonic development and tissue morphogenesis and function.

Here, we describe, for the first time, the generation of heparanase null mice. Southern blot analysis revealed specific integration of the vector, thereby deleting a part of the promoter and the first exon. This specific integration resulted in complete abolishment of heparanase mRNA expression and enzymatic activity, as revealed by PCR analysis applying 3 different pairs of primers and lack of heparanase enzymatic activity in blood samples and tissue extracts of the *Hpse-*KO mice. Generation of heparanase KO mice was further demonstrated by biochemical analysis revealing longer and more homogenous HS chains in *Hpse-*KO compared to *wt* mice. This molecular profile of HS is a mirror image of that found in heparanase over-expressing transgenic mice [36]. There were no significant differences in sulfation level and disaccharide composition.

HS structure and composition are known to affect embryonic development and tissue remodelling [45], [46]. Surprisingly, either shorter or longer HS chains failed to significantly affect embryonic development and life span of our *Hpse* transgenic or null mice, suggesting a large degree of flexibility in HS chain length in affecting organ development and tissue function. The fact that HS chains were longer and that there was no apparent degradation of HS in the *Hspe*-KO mice further supports the notion that heparanase is the predominant and likely only functional endoglycosidase capable of degrading HS.

Previous studies implicated heparanase in various normal and pathological processes ranging from bone remodelling, hair growth and kidney function to cancer metastasis, inflammation and angiogenesis [14], [19], [29], [47], [48], [49]. Heparanase is thought to act both as a hydrolytic enzyme, facilitating cell migration and release of ECM-resident bioactive factors, and as a signalling molecule, independent of its enzymatic activity [19], [50], [51]. Nevertheless, the *Hpse*-KO mice appeared normal, phenotypically and histologically, exhibiting a normal litter size and life expectancy. There were no detectable differences between the *Hpse-*KO and *wt* mice in platelet, kidney and liver functions, nor in their response to insults such as hepatectomy, delayed type hypersensitivity (DTH) reaction or wound healing (our unpublished results). Unexpectedly, we noticed an increased angiogenic response, both *ex-vivo* and *in-vivo*, and over branching of ducts in mammary glands of the *Hpse-*KO vs. their *wt* counterpart mice.

The lack of a major phenotype in the *Hpse-*KO mice could be attributed to changes in the expression levels of MMP family members, primarily MMP-2 and MMP-14, observed in the *Hpse-*KO mice. MMPs (i.e., MMP-2, MMP-14, MMP-9) degrade the ECM and, similar to heparanase, release bioactive molecules that are stored in the ECM, including, for example, pro-angiogenic factors such as FGF-2, VEGF and HGF [52], [53], [54]. MMPs, particularly stromolysin, are also involved in morphogenesis and over branching of the mammary gland [55]. It is therefore conceivable that MMPs may compensate for the lack of heparanase activity and hence mask some of the effects associated with its deficiency. In fact, at least 14 different MMP knockout mice have been generated, all of which survived to birth and exhibited notably subtle phenotypes [54]. This observation was attributed to the fact that MMPs have many overlapping substrates *in vitro*, resulting in a possible redundancy *in vivo*. Unlike the large number of MMPs, a single functional heparanase appears to be used by cells to degrade the HS side chains of HSPGs, making it an ideal target for gene disruption. The present study suggests that heparanase, despite of having a different type of substrate, may, in some aspects, share redundancy with MMP family members by virtue of its action on a common global substrate, namely the extracellular matrix and basement membrane.

The observed accumulation of β-catenin in *Hpse-*KO tissues and the increased occupation of the MMP-14 promoter by β-catenin, suggest a possible molecular pathway linking heparanase and MMPs. β-Catenin, previously implicated in regulating the expression of MMP-7 [56], [57], MMP-14 [38], MMP-2 and MMP-9 [39], is a central mediator in the canonical Wnt signaling cascade involved in developmental processes [58]. It is conceivable that ECM remodelling and tissue morphogenesis in the *Hpse*-KO mice are mediated by activation of the β-catenin pathway and the associated upregulation of MMPs.

Over-expression of heparanase in breast carcinoma cells (MDA-MB-231) was associated with a marked decrease in the expression of MMP family members, yielding a mirror image of the results obtained with the *Hpse-*KO mice. Notably, this effect was dependent on heparanase enzymatic activity. Thus, when the cells were transfected with mutant heparanase lacking enzymatic activity, there was no change in MMP expression. It should be noted that not all MMPs are upregulated in response to heparanase gene silencing. Thus, unlike liver and kidney, we observed, for example, a decrease in MMP-14 and MMP-25 in the mammary glands of *Hpse-*KO mice (not shown), suggesting that a tissue specific co-regulation of heparanase and MMPs should be taken into account. Similarly, it appears that in a given tissue some MMPs are upregulated while other can be downregulated in response to heparanase gene knock out. Co-regulation of heparanase and MMP-9 expression was recently demonstrated in myeloma cells [59]. In this system, however, transfection of heparanase into myeloma cells, or addition of recombinant active heparanase enhanced their expression of pro MMP-9 *in vitro* and knockdown of heparanase expression in *wt* myeloma cells reduced the level of MMP-9 expression. This effect is likely mediated by ERK signaling [59]. Regardless of the mode of action, it appears that heparanase may function as a master regulator of protease expression and activity within tissues and the tumor microenvironment during the course of tissue morphogenesis and tumor progression. The newly identified interrelation between the two types of distinct ECM degrading enzymes, MMPs which degrade proteins and heparanase degrading HS saccharide chains, suggests a co-regulatory mechanism that affects the expression of both types of enzymes. It appears that a common cellular sensor monitors the need for ECM remodelling and regulates the balance between heparanase and MMP expression. It is conceivable that this putative sensor is activated upon release from the ECM depot, and, as proposed in our study, regulates MMP expression via the β-catenin pathway.

Unexpectedly, both overbranching of mammary glands and the enhanced angiogenic response observed in the *Hpse*-KO mice resembled the phenotype seen in heparanase over-expressing transgenic mice. This presumed incongruity could be explained by the fact that both mammary morphogenesis and the angiogenic process involve massive ECM remodelling. In both the *Hpse-*KO and *hpa-*tg mice, regulation of heparanase expression levels appears to be compromised by up- and down- regulation of MMPs, respectively. The overall end morphological appearance is the net result of the balance and co-regulation of ECM-degrading enzymes such as heparanase and MMPs.

Heparanase has been previously shown to affect gene expression. We have demonstrated, for example, that over-expression of the heparanase gene and even exogenous addition of recombinant heparanase, up-regulate the expression of VEGF [51] and tissue factor [60]. Applying the *hpa-*tg and *Hpse-*KO mouse models, we have demonstrated up-regulation [60] and down-regulation (our unpublished results) of tissue factor levels, *in vivo.* Preliminary gene array studies indicate that other genes are affected, as well. It has also been demonstrated that nuclear translocation of heparanase is associated with induction of gene expression and cell differentiation [61], [62]. It is, therefore, not surprising that heparanase gene silencing and/or over expression affect the expression of other ECM-degrading enzymes such as MMPs.

Numerous studies emphasize the involvement of MMPs and heparanase in disease situations associated with cancer metastasis, angiogenesis and inflammation [14], [15], [19], [52], [54]. Hence, the observed regulatory association between heparanase and members of the MMP superfamily may have important clinical implications. A major effort is invested in developing specific inhibitory molecules and strategies targeting heparanase [63], [64] and MMPs [65], [66]. Clinical trials evaluating the therapeutic potential of MMP inhibitors yielded disappointing results, in part due to redundancy among family members, compensating for the inhibited target enzyme. The observed up-regulation of MMPs in response to heparanase gene knockout favours a combined approach in which heparanase and MMP inhibiting compounds are co-administered. 

Our generation of viable *Hpse*-KO mice lacking major visible abnormalities is regarded as a good argument for the development of heparanase-based drugs, presumably with little or no side effects. However, we cannot exclude the possibility that subtle, unidentified phenotypes may cause some side effects, depending on the drug and the target tissue.
